# Management of ovarian immature teratoma grade‐II in pregnancy, two cases report and literature review

**DOI:** 10.1002/ccr3.4456

**Published:** 2021-07-10

**Authors:** Fatemeh Homaei Shandiz, Ali Emadi Torghabeh

**Affiliations:** ^1^ Cancer Research Center Mashhad University of Medical Sciences Mashhad Iran; ^2^ Faculty of Medicine Department of Radiotherapy and Oncology Mashhad University of Medical Sciences Mashhad Iran

**Keywords:** chemotherapy, ovarian immature teratoma grade 2, pregnancy

## Abstract

Postoperative chemotherapy during pregnancy after first trimester is essential for patients with initial disease stage 1, grade 2 ovarian immature teratoma and it associates with lower disease progression and recurrence.

## Graphical abstract

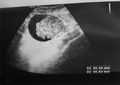

## 1 INTRODUCTION

The occurrence of immature ovarian teratoma is a rare condition during pregnancy. Considering the difficulties of surgery and adjuvant chemotherapy in high‐grade cases during the pregnancy, its management is controversial. Here, two cases of grade‐II ovarian immature teratomas in two pregnant women along with their management and outcomes were presented.

Ovarian germ cell tumors are considered as a rare female malignancy accounting for less than five percent of all ovarian tumors with an incidence rate of 0.34 per 100,000 women per years.^1^, ^2^ The occurrence of this ovarian tumor during the pregnancy is extremely scarce, and currently, there are not enough data on its clinical presentation, appropriate diagnostic work‐up, and management except some case reports.

The immature ovarian teratomas of ovaries are originated from the three germ layers (endo‐, meso‐ and ectoderm). Histological tumor grades, based on the amount of immature neuroepithelium within the tumoral tissue, determine the prognosis of patients directly.^3^, ^4^

In nonpregnant patients, treatments mainly depend on the surgical resection of the primary tumor with adjuvant chemotherapy in high‐grade tumors. However, during the pregnancy, the management of these tumors is controversial considering the difficulties of surgery and also adjuvant chemotherapy in this situation.

Here, two cases of grade‐II ovarian immature teratoma in two pregnant women along with their management and outcomes were presented.

## 2 CASE 1

**FIGURE 1:**
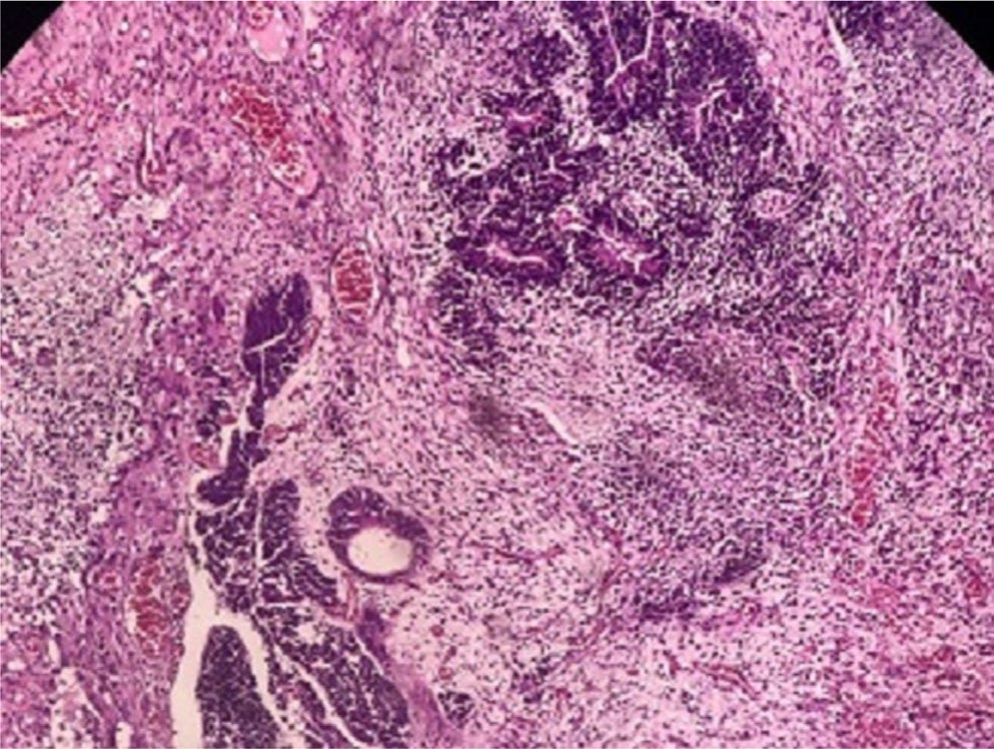
Immature component of teratoma including neuroepithelial elements with rosette formation

The patient was a primigravid 21‐year‐old woman whose complaint was abdominal pain in the sixth week of pregnancy. She has no specific past medical history, and the familial history was negative. The patient underwent abdomen‐pelvic ultrasonography showing a live embryo at the age of 6–7 weeks along with a right ovarian cyst at the size of 140 × 150 mm consists of the solid component. Laboratory assessments were normal. The patient underwent laparotomy and right ovarian cystectomy. Pathological assessment of the ovarian tumor revealed a yellow 150 × 140 mm solid cystic lesion on the right ovary that microscopically there were embryonic tissues from all three cell lines including adipose, cartilage, and neurogenic connective tissues, confirming the diagnosis of grade‐II immature teratoma (Figure 1). Then, the patient referred and evaluated in a multidisciplinary team (MDT) recommended a metastasis wok‐up using a chest x‐ray (CXR) by shielding of abdomen and pelvis and initiation of adjuvant chemotherapy by BEP regimen (bleomycin 30 unit on days 1, 8, 15 plus etoposide 100 mg/m^2^ days 1–5 plus cisplatin 20 mg/m^2^ days 1–5) while the pregnancy continued. In addition, for comprehensive staging, the MDT recommended that complete surgical staging by a fertility‐preserving approach be considered after delivery.

Results of the prechemotherapy evaluation were normal. At the 15 weeks 6‐day gestational age, chemotherapy by BEP regimen every 3 weeks began. After the third cycle of chemotherapy, whereas the age of the fetus was 26 weeks, the patient underwent cesarean section (C/S) due to gestational hypertension followed by eclampsia. During C/S, a complete surgical staging by a fertility‐preserving approach using the complete right oophorectomy and peritoneal fluid sampling was performed. Pathologic assessment of right ovary and peritoneal fluid was negative for malignancy. The general condition of the patient was good, but unfortunately, a male newborn died due to immaturity. Thereafter, the patient followed in our clinic every 2–3 months. After 2 years of follow‐up, all clinic and paraclinical evaluations including tumor markers, imaging of chest, and abdomen‐pelvic are normal. Besides, she got pregnant 15 months after the completion of treatment and now she has a 1‐year‐old baby.

## 3 CASE 2

**FIGURE 2:**
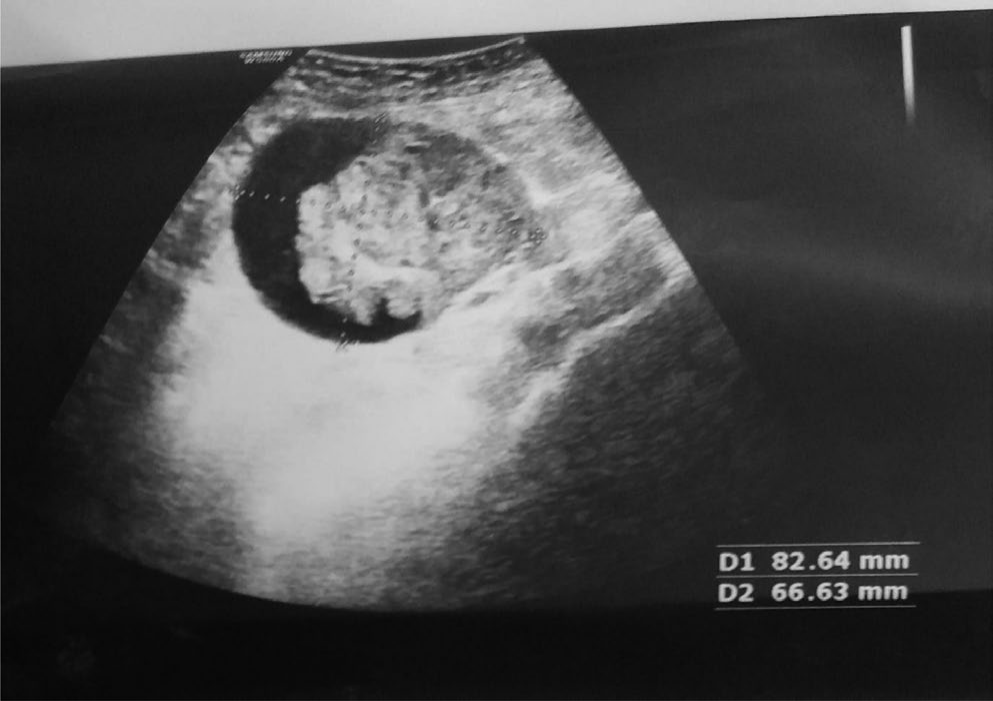
Ovarian cystic mass with solid component

**FIGURE 3:**
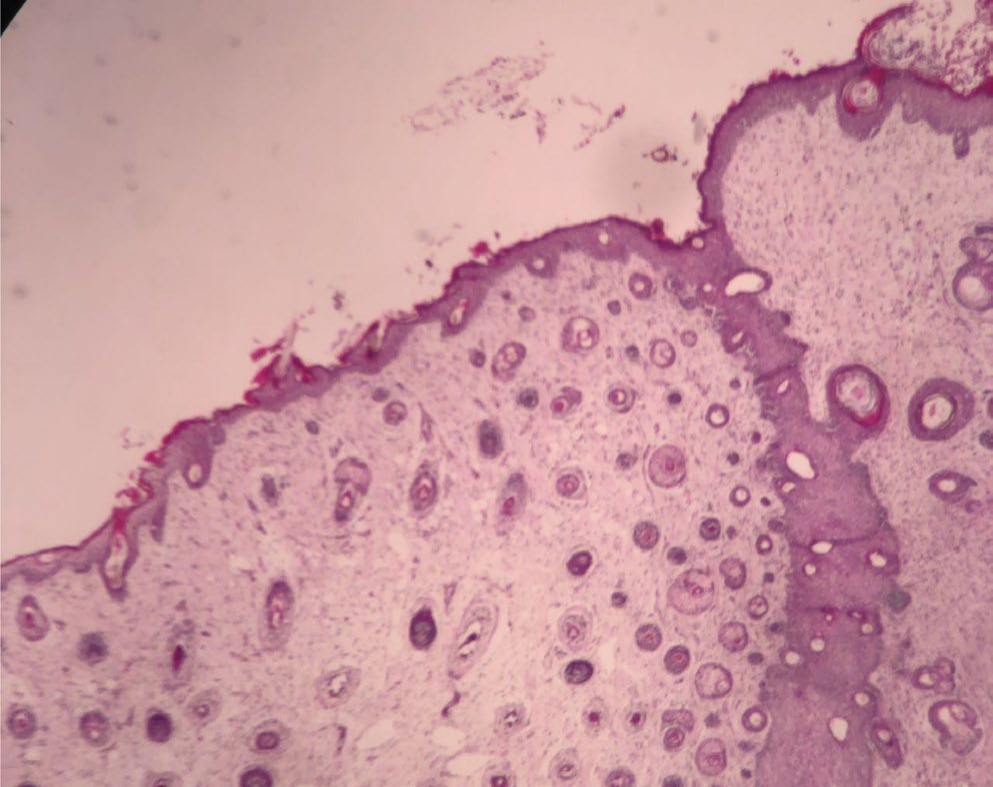
Mature ectodermal component including squamous epithelium and hair follicles

The patient was a multigravid 2, live 1, 29‐year‐old woman complaining of abdominal pain in the second month of pregnancy. The abdomen‐pelvic ultrasonography reported an intrauterine 7‐week live embryo along with an 82 × 64 mm left adnexal cystic mass consisted of a 58* 41 mm solid component (Figure 2). After 1 month, she underwent laparoscopic resection of the left ovarian mass. Pathologic assessment of ovarian tumor revealed immature teratoma, grade 2 (Figure 3). The patient was evaluated in an MDT in our department, and metastasis work‐up by a fetus protected CXR, and initiation of adjuvant chemotherapy using BEP regimen and completion of pregnancy were recommended.

The CXR was normal. However, the patient revoked her consents to perform adjuvant chemotherapy until the delivery. At the delivery, she underwent C/S, resection of the abdominal mass, and a male baby was born. The surgeon reported multiple tumoral implants in the bowel meso and omentum along with hemorrhagic ascites. Pathologic assessment of the resected mass revealed "metastatic immature teratoma, grade 2 with positive ascites fluid for the presence of malignant cells." After the surgery, there were an increased level of alpha‐fetoprotein (AFP); 117 U/ml (0.2–8.5) and cancer antigen 125 (CA125); 146 U/ml (0–35) with a normal human chorionic gonadotropin (BHCG). Three cycles of chemotherapy based on BEP regimen were administered, and postchemotherapy evaluation showed a significant decrease in the level of AFP and CA125 (7.3 U/ml and 14 U/ml, respectively). A whole‐body CT scan showed evidence of peritoneal seeding and implants around the liver and in sub‐diaphragmatic, paracolic, and pelvic regions and also two bilateral supra‐diaphragmatic lymphadenopathies. The chemotherapy was continued for two more cycles, and the laboratory assessment showed a continuum reduction of AFP and CA125 (4.9 and CA125 = 19.9, respectively). Despite the reduction of tumor markers, the CT scan showed evidence of radiologic disease progression. Based on MDT recommendation, the patient underwent a needle biopsy from supra‐diaphragmatic lymphadenopathies showing mature teratoma on the pathological evaluation. Considering the low tumor marker salvage surgical resection of all tumoral masses was recommended by MDT; however, the surgeon reported diffuse tumoral seeding in the abdominal wall and considered the lesion inoperable. Subsequently, the patient received six cycles of paclitaxel (175 mg/m^2^) + carboplatin (AUC = 5) every 3 weeks. After 4 years of diagnosis, the patient was progression‐free.

## 4 DISCUSSION

Pure immature teratomas, first characterized by Norris et al. in 1976, are rare germ cell tumors that involve three germ layers with at least one of the components having an immature appearance.^2^, ^5^ Survival is determined by the size and stage of teratomas, but the grade of the primary tumor is the most important determinant of the likelihood of extra ovarian spread and for the subsequent course. Grading is based on the amount of immature neural tissue.^2^ Zhang R. et al reported a 5‐year survival rate as 92% for immature teratoma based on 63 (nonpregnant) cases with immature teratoma among 145 malignant ovarian germ cell tumor cases, and histology, surgical approach, chemotherapy, and regimens were not predictive of 5‐year survival rates. Moreover, they concluded that fertility‐sparing treatment should be considered for ovarian germ cell tumors regardless of the FIGO stage.^6^

Young et al. showed that the prognosis for ovarian malignancies was not complicated by concurrent gestation if adequate treatment is administered timely.^7^ Management of ovarian tumors in pregnancy requires a multidisciplinary approach, and therapeutic decision should take into account histology, grade and stage of the tumor, and the pregnancy age.^8^ The prognosis for immature teratomas has improved due to the routine use of imaging during pregnancy and because of chemotherapy, and most ovarian cancers associated with pregnancy are detected by ultrasonography.^9^ The main presenting symptom for ovarian immature teratomas during pregnancy is adnexal mass followed by abdominal or pelvic pain. Some patients present with combined abdominal pain and mass, and some patients are asymptomatic and detect by routine ultrasonography and by elevated serum AFP.^10^ Both our patients also refer with the primary complaint of abdominal pain. Given malignant ovarian germ cell tumors are usually unilateral, except advanced stage cases with metastasis to the contralateral ovary, unilateral salpingo‐oophorectomy with preservation of the contralateral ovary and uterus is appropriate for treatment of most cases. If metastatic disease is detected during surgery, cytoreductive surgery is recommended. Second look laparotomy for germ cell tumors is controversial, and if inadequate staging was present at the first operation, second‐look surgery or CT should be considered.^11^ The poor prognosis of malignant germ cell tumors treated by surgery alone indicates a need for adjuvant chemotherapy.^4^ The risk of major malformation during the first trimester of pregnancy is 10% for single‐agent chemotherapy and 25% for combination chemotherapy.^5^, ^6^, ^8^ Afterward, the second trimester seems safer for chemotherapy.

Based on NCCN guideline, patients affected by stage 1, grade 1 ovarian immature teratoma, have an excellent prognosis without adjuvant chemotherapy; therefore, these patients only observe postoperatively. The patients with stage1 and grade 2 or 3 of immature teratoma and the patients with stages 2 or 3 at any grades need postoperative chemotherapy by BEP regimen for 3–4 cycles every 3 weeks. After completion of chemotherapy, if you have complete clinical responses in laboratory and imaging studies, observation is recommended. But if the imaging studies reveal residual disease with normal tumor marker level, both surgery and observation are advisable and ultimately, if the tumor marker level is high and the imaging studies reveal residual disease too, salvage or high‐dose chemotherapy is recommended.

Although there is limited experience for using this regimen during pregnancy,^12^, ^13^, ^14^, ^15^, ^16^ the BEP treatment has been associated with ventriculomegaly, transient neonatal neutropenia, and bilateral sensorineural hearing loss. Considering the co‐occurrence of blood hypertension and eclampsia during administration of BEP regimen, more data are mandatory for safety profile of this regimen.

## 5 CONCLUSION

Based on our two mentioned cases and the other data, it seems that postoperative chemotherapy during pregnancy after the first trimester is essential for patients with initial disease stage 1, grade 2 ovarian immature teratoma and it associates with lower disease progression and recurrence.

## CONSENT STATEMENT

Published with written consent of the patient.

## Data Availability

Data sharing was not applicable to this article as no datasets were generated or analyzed during the current study.
